# Cross-sectional and prospective relationship between occupational and leisure-time inactivity and cognitive function in an ageing population: the European Prospective Investigation into Cancer and Nutrition in Norfolk (EPIC-Norfolk) study

**DOI:** 10.1093/ije/dyaa067

**Published:** 2020-07-06

**Authors:** Shabina A Hayat, Robert Luben, Nick Wareham, Kay-Tee Khaw, Carol Brayne

**Affiliations:** d1 Department of Public Health and Primary Care, Institute of Public Health, University of Cambridge, Cambridge, UK; d2 MRC Epidemiology Unit, University of Cambridge School of Clinical Medicine, Cambridge, UK

**Keywords:** Ageing, cognitive function, physical inactivity, prospective cohort study

## Abstract

**Background:**

The current evidence for higher physical activity and better cognitive function and lower risk of dementia is strong but not conclusive. More robust evidence is needed to inform public-health policy. We provide further insight into discrepancies observed across studies, reporting on habitual inactivity including that during work.

**Methods:**

We examined cross-sectional and prospective relationships of physical inactivity during leisure and occupation time, with cognitive performance using a validated physical-activity index in a cohort of 8585 men and women aged 40–79 years at baseline (1993–1997) for different domains using a range of cognitive measures. Cognitive testing was conducted between 2006 and 2011 (including a pilot phase 2004–2006). Associations were examined using multinomial logistic-regression adjusting for socio-demographic and health variables as well total habitual physical activity.

**Results:**

Inactivity during work was inversely associated with poor cognitive performance (bottom 10th percentile of a composite cognition score): odds ratio (OR) = 0.68 [95% confidence interval (CI) 0.54, 0.86], *P* = 0.001. Results were similar cross-sectionally: OR = 0.65 (95% CI 0.45, 0.93), *P* = 0.02. Manual workers had increased risk of poor performance compared with those with an occupation classified as inactive. Inactivity during leisure time was associated with increased risk of poor performance in the cross-sectional analyses only.

**Conclusions:**

The relationship between inactivity and cognition is strongly confounded by education, social class and occupation. Physical activity during leisure may be protective for cognition, but work-related physical activity is not protective. A greater understanding of the mechanisms and confounding underlying these paradoxical findings is needed.

**Key Messages**

- The evidence for physical activity to be a protective factor for cognitive decline and dementia is strong but not conclusive.
- This prospective study is the first to investigate the relationship between habitual physical inactivity during leisure and work time (combined and separately) with cognitive function from individuals from a wide range of socio-economic backgrounds and education.
- Physical inactivity at work was inversely associated with poor cognitive performance. Manual workers (with higher work physical activity) had almost three times the risk of poor performance compared with those reporting to have an inactive or sedentary occupation.
- This study shows a differential association between cognition and physical inactivity during work and leisure, and suggests that this association may be attributed to confounding by education, occupation and social class.

## Introduction

There is an increasing interest in the potential role of modifiable factors in preventing or delaying the onset of dementia.^1^ Physical inactivity and sedentary behaviour (independently of physical activity) have been reported to be risk factors for major health conditions,^2^ including cognitive impairment.^3^

The available evidence for future public-health strategies on how to best manage or prevent cognitive decline, impairment and dementia has shown physical activity to be predominantly, but not consistently, beneficial, with mixed evidence from observational studies.^4–6^ The reasons for these discrepancies are unclear,^7^ but may be partly due to the heterogeneity and limitations in the methodologies across studies. This includes differences in follow-up time,^8^ low power with insufficient sample size, differences in population characteristics^9–11^ and the variability in the way in which the exposure (physical activity) and the outcome (cognition) are measured and defined across studies.

Most studies have focused on moderate and severe cognitive impairment, including dementia, with far less on the relationship of physical activity and milder cognitive dysfunction. Cross-sectional studies with short follow-up times cannot distinguish causal effects from reverse causation and confounding is an issue highlighted as a limitation in observational studies.^5^^,^^8^ Experimental studies examining the influence of structured physical activity on enhancing cognitive function have also been inconsistent.^12^

Studies cited in the literature have differed in methodology, but have predominantly used leisure-time activity,^6^^,^^8^ with few examining work-based physical activity.^4^^,^^5^ Although leisure-time activity has been associated with better cognition,^9^ work-related physical activity has shown no relationship,^4^ or even the contrary, with lower socio-economic groups and manual occupations with higher physical activity showing greater risk of dementia and cognitive impairment.^5^^,^^13^ To advise on public-health strategies for maintaining cognition in later life for all in society, we need a better understanding of discrepancies in the existing evidence base.

We examine the cross-sectional and prospective relationship between physical inactivity and cognitive performance (in terms of both poor and high performance), in older men and women from a wide range of socio-economic backgrounds and education. We present findings on habitual inactivity including work and leisure time, using a simple pragmatic validated physical-activity scale.

## Methods

### Study population

The European Prospective Investigation of Cancer (EPIC) in Norfolk (EPIC-Norfolk) is a prospective cohort study of lifestyle factors and disease. Over 25 000 community-dwelling men and women (40–79 years old) were recruited at inception (1993–1997) from GP registers in Norfolk (UK). Participants completed a health and lifestyle questionnaire and underwent a clinical examination.^14^ Cognitive assessment was introduced at the third health examination (3HC) between 2006 and 2011 with a preceding pilot phase between 2004 and 2006 (participants aged 48–92 years). Participants had no overt cognitive problems. The full assessment was a comprehensive 3-hour examination, which included tests assessing different domains of cognitive function. Pilot data were included. Detailed descriptions have been published elsewhere.^14–18^

The EPIC-Norfolk core study was approved by Norwich Committee in 1992 (REC Ref: 98CN01). The 3HC was approved by the Norfolk Local Research Ethics Committee (05/Q0101/191) and East Norfolk and Waveney NHS Research Governance Committee (2005EC07L). Participants gave signed informed consent.

### Assessment of cognition

The EPIC-Norfolk cognition battery consisted of seven tests, assessing performance across different cognitive domains giving a total of eight separate cognitive measures. These tests have been described in detail^18^; they are summarized in Box 1.

**Box 2:** List of cognitive tests used in the EPIC-Norfolk

	Name of test	Predominant ability measured by test (description of score)
1	A shortened version of the Extended Mental State Exam (SF-EMSE)	Global function (continuous score)
2	Hopkins Verbal Learning Test (HVLT)	Verbal episodic memory (continuous score)
3	Cambridge Neuropsychological Test Automated Battery Paired Associates Learning Test. First trial Memory Score (CANTAB-PAL-FTMS)	Non-verbal episodic memory (continuous score)
4	PW Letter Cancellation Task (PW-Accuracy Score)	Attention (continuous score)
5	Event and Time Based Task (prospective memory)	Prospective memory (categorical score, success or fail)
	Visual Sensitivity Test (VST)	Simple and complex visual processing speed measured in milliseconds (continuous score)
6	VST-Simple	
7	VST-Complex	
8	Shortened version of the National Adult Reading Test (short-NART)	Reading ability and crystallized intelligence (continuous score)

### Assessment of physical activity

Total (habitual) physical activity was assessed using two questions (Supplementary Appendix 1, available as Supplementary data at *IJE* online). The first referred to usual occupational physical activity and the second on the amount of time spent in hours per week in both winter and summer on cycling and other exercise. A simple four-category physical-activity index was derived based on the level of activity during occupation and leisure time (Supplementary Appendix 2, available as Supplementary data at *IJE* online). The questionnaire was validated as a measure of physical-activity energy expenditure against individually calibrated heart-rate monitoring^19^ and was shown to predict total mortality and cardiovascular-disease incidence.^20^

### Covariates

Education (the highest level attained) and social class were derived from the baseline questionnaire. Education was categorized into three groups: (i) no qualification (not completing school to age 16), (ii) completing school to age 16 or 18 years (O or A level) and (iii) educated to graduate level (a degree or equivalent) or above. Social class was classified according to the Registrar General’s occupation-based classification scheme.^21^ These were then grouped into ‘non-manual’ and ‘manual’. Smoking status and alcohol intake (units/week) were obtained from questionnaires from baseline and close to the time of cognitive testing. Alcohol units were categorized into three groups: 0 Units, 1–14 Units and >14 Units. Age was categorized into 5-year age bands.

### Analyses

Descriptive analysis of cognitive scores by physical-activity category revealed a non-linear relationship. Associations were examined using approximate percentile cut-offs rather than the continuous cognition score. Participants were classified into three groups based on their scores, creating a three-level categorical variable for each of the cognitive measures. The lowest level (1) corresponded to poor performance (defined as obtaining a score less than a cut-off point corresponding to approximately the 10th percentile of the population distribution). The highest level (3) corresponded to high performance (defined as obtaining a score greater than a cut-off point corresponding to approximately the 90th percentile of the population distribution). The remaining were those within the 11th–89th percentiles, the standard level (2). For prospective memory, where participants either succeeded or failed the task, those who failed were assigned to the poor-performance group and those who succeeded to the standard level. To address the limitation of multiple testing, we created a composite score (EPIC-COGComp), representing general cognition underlying all the cognitive functions assessed (Supplementary Appendix 3, available as Supplementary data at *IJE* online). A three-level categorical variable was created for EPIC-COGComp for poor, high and standard group levels as for the cognitive measures individually.

The physical-activity index was examined in three different ways: first, the four-point index was used to examine the characteristics of the population by category of physical activity both at baseline and at the 3HC; second, by dichotomizing the index into ‘inactive’ and ‘active’; and, finally, associations were examined using the composite score and the dichotomized activity index for occupation and leisure-time components separately.

**Figure 1:**
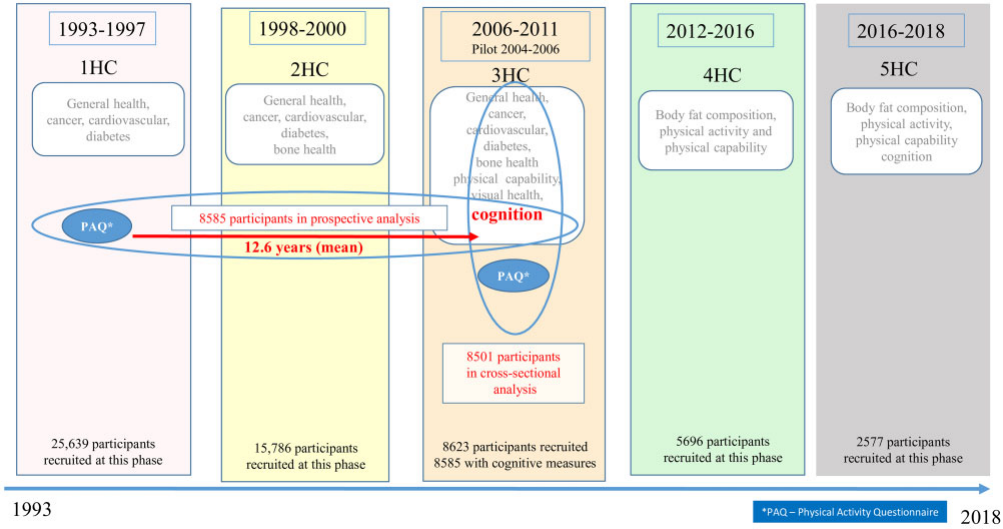
Timeline of the EPIC-Norfolk study showing the five health-check phases over 25 years, the main area of interest for each phase and the number of participants recruited to study at that time point. Cognitive measures from the Third Health Check (3HC) were used in this analysis. Physical-activity measures taken from baseline (1HC for prospective analysis) and 3HC (for cross-sectional analysis). 1HC, First Health Check; 2HC, Second Health Check; 3HC, Third Health Check; 4HC, Fourth Health Check; 5HC, Fifth Health Check; PAQ, Physical Activity Questionnaire.

Associations between cognition (poor and high performance) and physical inactivity at the time of cognitive testing at 3HC (cross-sectional) and at baseline (prospective) were examined using multinomial logistic regression. Figure 1 is a timeline of the study, presenting the two time points used in the analyses. Associations were assessed adjusting for age at time of cognitive testing (per 5 years) and sex (Model 1), adding education and social class (Model 2), extending the models to include prevalent disease (Model 3) and, finally, adjusting further for total habitual activity (Model 4) in the separate work and leisure analyses. Education, social class, physical activity, smoking and prevalent disease were all treated as categorical variables in the analysis and age per 5 years entered as continuous.

We also examined possible interaction with education and work-related activity and stratified by education group before calculating adjusted odd ratios for work and leisure. Sensitivity analyses were conducted (i) by assigning participants with missing data to the poor-performance group and (ii) by grouping participants into approximate quartiles (rather three levels) of cognition scores.

## Results

From baseline to time of cognitive testing (3HC), the mean follow-up time was 12.6 years (SD = 2.0). There were 8585 participants with cognitive measures, resulting in 8501 participants in the cross-sectional and 8585 in the prospective analyses, respectively. There were slightly fewer in the cross-sectional analyses due to some missing physical-activity data. Those invited for the 3HC (*N* = 18 382) but did not attend were more likely to be older, with higher self-reported heart-attack, stroke and diabetes prevalence. Non-attenders were also more likely to have no qualifications and be in the lower socio-economic groups (Supplementary Table 1, available as Supplementary data at *IJE* online).

Tables 1 and 2 show the characteristics (unadjusted) of the participants by habitual physical-activity category for men and women at baseline and the 3HC, respectively. At baseline, for men, a greater proportion of the moderately inactive and inactive were educated to graduate level and were from higher socio-economic groups. There were also more current smokers compared with those in the other levels of activity. For women, the inactive groups had fewer individuals in the higher educated and socio-economic groups, although, as with men, more current smokers. Inactive individuals (for both men and women) were more likely to be older, with men reporting higher rates of diabetes and women having higher rates of depression. At 3HC (Table 2), the majority of individuals reported being retired from their main occupation and were more likely to be inactive. Inactive men and women had higher rates of heart attack and stroke, and more were non-drinkers and current smokers. There were no clear differences between the level of physical activity (for baseline or 3HC) and the score for any of the cognitive tests.

**Table 1:** Baseline (1993–1997) characteristics of the 8585 EPIC-Norfolk men and women with cognitive data collected at the third health-check phase (3HC 2006–2011, including data from pilot 2004–2006) by level of physical activity

		Level of activity as reported at baseline by men				
Characteristics at	All	Inactive	Moderately inactive	Moderately active	Active	*P*-value
baseline	*N* = 3841	*N* = 880	*N* = 1001	*N* = 973	*N* = 987	
**Mean (SD)**						
Age	56.4 (7.9)	58.1 (8.0)	56.3 (8.0)	56.1 (7.7)	55.1 (7.5)	<0.001
Frequencies, % (*N*)						
**Level of education**						
No qualification	22.1 (847)	22.9 (201)	19.2 (192)	19.9 (194)	26.3 (260)	<0.001
O or A level	57.8 (2221)	59.5 (523)	52.5 (526)	60.4 (588)	59.2 (584)	
Graduate level	20.1 (772)	17.6 (155)	28.3 (283)	19.6 (191)	14.5 (143)	
**Social class**						
Professional	9.5 (364)	11.1 (97)	14.6 (145)	7.7 (74)	4.9 (48)	<0.001
Managerial	42.7 (1629)	47.0 (411)	47.6 (473)	42.3 (408)	34.4 (337)	
Skilled non-manual	12.2 (466)	16.2 (142)	15.8 (157)	9.9 (96)	7.2 (71)	
Skilled manual	22.7 (865)	14.4 (126)	13.1 (130)	27.4 (264)	35.2 (345)	
Semi-skilled	11.0 (418)	9.8 (86)	7.8 (78)	10.8 (104)	15.3 (150)	
Non-skilled	1.9 (72)	1.5 (13)	1.1 (11)	2.0 (19)	3.0 (29)	
**Co-morbidities**						
Heart attack	2.6 (101)	3.6 (32)	2.4 (24)	2.6 (25)	2.0 (20)	0.2
Stroke	0.7 (27)	0.8 (7)	0.9 (9)	0.9 (9)	0.2 (2)	0.2
Cancer	2.8 (106)	2.5 (22)	2.6 (26)	3.9 (38)	2.0 (20)	0.07
Diabetes	1.4 (54)	2.3 (20)	1.4 (14)	1.8 (18)	0.2 (2)	0.001
Depression	8.6 (329)	9.2 (81)	9.5 (95)	8.0 (78)	7.6 (75)	0.4
** Alcohol**						0.2
** (units/week)**						
0	5.9 (227)	5.7 (50)	5.0 (50)	6.7 (65)	6.3 (62)	
≤14 Units	68.3 (2614)	71.1 (619)	67.4 (672)	68.3 (663)	66.9 (660)	
>14 Units	25.7 (984)	23.2 (202)	27.6 (275)	25.0 (243)	26.8 (264)	
**Smoking status**						
Never	41.4 (1586)	36.9 (324)	44.6 (445)	41.0 (397)	42.6 (420)	0.04
Former	49.6 (19.1)	53.0 (465)	47.4 (473)	49.5 (480)	49.0 (483)	
Current	8.9 (342)	10.0 (88)	8.0 (80)	9.5 (92)	8.3 (82)	
**Mean Cognitive Test Score at 3HC (SD)**						
SF-EMSE	32.5 (3.3)	32.2 (3.7)	32.9 (2.8)	32.6 (3.4)	32.3 (3.4)	<0.001
HVLT	23.9 (5.6)	23.6 (5.8)	24.2 (5.7)	23.9 (5.6)	23.7 (5.4)	0.1
PAL-FTMS	15.4 (4.3)	15.3 (4.3)	15.4 (4.5)	15.6 (4.2)	15.2 (4.3)	0.3
PW-Accuracy	12.3 (5.6)	12.3 (6.0)	12.7 (5.9)	12.4 (6.1)	12.0 (6.3)	0.08
VST-simple^c^	657.7 (161.6)	658.8 (175.2)	656.4 (161.9)	654.9 (151.1)	661.0 (158.8)	0.9
VST-Complex^c^	2227.5 (422.9)	2267.8 (499.4)	2235.5 (440.4)	2200.7 (375.4)	2210.1 (369.2)	0.01
NART^c^	17.9 (10.3)	16.4 (10.0)	16.2 (9.8)	18.6 (10.3)	20.3 (10.5)	<0.001
Comp. Score	7.7 (1.8)	7.7 (1.8)	7.8 (1.8)	7.7 (1.8)	7.4 (1.8)	0.001
Pros.Mem % failed (*N*)	22.3 (838)	20.6 (176)	21.6 (211)	23.0(219)	24.1 (232)	0.3

**Table 1:** Continued

		Level of activity as reported at baseline by women				
Characteristics at baseline	All	Inactive	Moderately inactive	Moderately active	Active	*P*-value
	*N* = 4744	*N* = 976	*N* = 1577	*N* = 1230	*N* = 961	
**Mean (SD)**						
Age	55.1 (7.7)	56.9 (8.2)	55.4 (7.9)	54.4 (7.4)	53.8 (7.1)	<0.001
Frequencies, % (*N*)						
** Level of education**						
No qualification	29.6 (1404)	31.5 (307)	28.2 (445)	29.4 (362)	30.2 (290)	<0.001
O or A level	54.8 (2598)	56.8 (554)	55.5 (876)	51.5 (634)	55.6 (534)	
Graduate level or above	15.6 (741)	11.7 (114)	16.2 (256)	19.0 (234)	14.3 (137)	
**Social class**						
Professional	8.2 (384)	5.6 (54)	9.0 (141)	9.2 (112)	8.1 (77)	<0.001
Managerial	39.8 (1869)	35.5 (342)	41.1 (642)	41.93 (511)	39.5 (374)	
Skilled non-manual	19.1 (898)	23.5 (226)	20.4 (319)	17.3 (211)	15.0 (142)	
Skilled manual	18.8 (883)	21.2(204)	17.7 (277)	17.0 (207)	20.6 (195)	
Semi-skilled	11.3 (532)	11.0 (106)	10.1 (158)	11.6 (141)	13.4 (127)	
Non-skilled	2.7 (125)	3.2 (31)	1.5 (24)	3.0 (37)	3.5 (33)	
**Co-morbidities**						
Heart attack	0.6 (27)	0.4 (4)	0.6 (9)	0.6 (7)	0.7 (7)	0.8
Stroke	0.5 (23)	0.7 (7)	0.5 (8)	0.4 (5)	0.3 (3)	0.6
Cancer	5.7 (268)	6.8 (66)	5.2 (82)	5.7 (70)	5.2 (50)	0.4
Diabetes	0.7 (31)	0.5 (5)	0.9 (14)	0.4 (5)	0.7 (7)	0.4
Depression	19.5 (921)	22.7 (221)	19.4 (305)	18.1 (222)	18.0 (173)	0.03
**Alcohol (units/week)**						0.1
0	11.4 (536)	13.6 (131)	10.2 (160)	10.4(128)	12.2 (117)	
≤14 Units	80.8 (3812)	78.9 (758)	82.0 (1288)	81.0 (993)	80.5 (773)	
>14 Units	7.8 (369)	7.5 (72)	7.8 (122)	8.6 (105)	7.3 (70)	
**Smoking status**						
Never	60.9 (2877)	58.0 (563)	60.4 (947)	63.2 (774)	61.9 (593)	0.02
Former	30.2 (1428)	30.9 (300)	30.8 (483)	28.1 (344)	31.4 (301)	
Current	8.9 (418)	11.1 (108)	8.9 (139)	8.7 (107)	6.7 (64)	
** Mean Cognitive Test Score at 3HC (SD)**						
SF-EMSE	32.7 (3.0)	32.6 (3.1)	32.7 (3.1)	32.8 (2.8)	32.6 (3.0)	0.2
HVLT	26.04 (5.5)	25.7 (5.9)	26.1 (5.6)	26.4 (5.3)	25.9 (5.3)	0.04
PAL- FTMS	15.8 (4.2)	15.7 (4.2)	15.8 (4.3)	15.9 (4.2)	15.8 (4.2)	0.6
PW-Accuracy	13.8 (5.9)	13.4 (6.3)	14.0 (5.8)	14.0 (5.9)	13.8 (5.8)	0.08
^a^VST-simple^b^	668.7 (169.9)	690.3 (212.0)	662.1 (155.7)	663.3 (148.5)	664.6 (168.5)	0.001
^a^VST-Complex^b^	2172.3 (432.5)	2205.4 (481.4)	2163.7 (455.5)	2173.3 (403.1)	2151.3 (371.2)	0.07
NART ^b^	16.6 (9.5)	16.8 (9.7)	16.1 (9.2)	16.4 (9.4)	17.6 (9.6)	0.001
Composite score	8.1 (1.8)	8.0 (2.0)	8.2 (1.8)	8.1 (1.8)	8.0 (1.7)	0.3
Pros.Mem % failed (N)	15.9 (738)	16.1 (154)	15.6 (241)	15.5 (187)	16.5 (156)	0.9

A, Advanced; HVLT, Hopkins Verbal Learning Test; *N*, number; NART, National Adult Reading Test; O, Ordinary; PAL-FTMS, Paired Associates Learning, First Trial Memory Score; Pros.Mem, Prospective Memory; SD, standard deviation; SF-EMSE, Short Form Extended Mental State Exam; VST, Visual Sensitivity Test.

a Reaction time measured in milliseconds.

b Higher scores indicate lower performance.

**Table 2:** Characteristics of EPIC-Norfolk men (*N* = 3786^a^) and women (*N* = 4680^a^) at time of cognitive testing (3HC Phase, 2006–2011, including data from pilot 2004–2006) by level of physical activity

		Men				
	All	Inactive	Moderately inactive	Moderately active	Active	*P*-value
	*N* = 3841^a^	*N* = 1410	*N* = 952	*N* = 712	*N* = 712	
**Mean (SD)**						
Age	69.4 (8.1)	72.0 (7.9)	69.2 (7.9)	67.4 (7.8)	66.4 (7.3)	0.01
Frequencies, % (*N*)						
Retired from main occupation	75.9 (2842)	81.7 (701)	77.1 (749)	76.5 (727)	69.0 (665)	<0.001
**Co-morbidities**						
Heart attack	5.2 (198)	7.1 (100)	4.1 (39)	4.2 (30)	3.5 (25)	<0.001
Stroke	3.0 (116)	4.7 (66)	2.2 (21)	2.1 (15)	1.5 (11)	<0.001
Cancer	7.2 (276)	7.5 (106)	7.8 (74)	6.7 (48)	6.2 (44)	0.6
Diabetes	3.9 (151)	5.2 (74)	3.0 (29)	4.1 (29)	2.4(17)	0.01
Depression	14.0 (536)	13.1 (185)	15.9 (151)	14.5 (103)	12.4(88)	0.2
**Alcohol (units/week)**						
0	22.2 (824)	27.3 (375)	18.5 (173)	20.5 (144)	18.9 (132)	<0.001
≤14 Units	59.2 (2193)	55.6 (764)	62.6 (585)	60.6 (425)	59.9 (419)	
>14 Units	18.6 (690)	17.0 (234)	18.8 (176)	18.8 (132)	21.2 (148)	
Current smokers, % (*n*)	4.2 (158)	4.8 (68)	3.9 (37)	3.2 (23)	4.2 (30)	
**Mean Cognitive Test Score (SD)**						
SF-EMSE	32.5 (3.3)	32.0 (3.7)	32.9 (3.0)	32.8 (2.9)	32.7 (2.8)	<0.001
HVLT	23.9 (5.6)	23.0 (5.8)	24.3 (5.6)	24.5 (5.4)	24.5 (5.1)	<0.001
PAL- FTMS	15.4 (4.3)	14.7 (4.4)	15.6 (4.3)	16.1 (4.1)	15.8 (4.1)	<0.001
PW-Accuracy	12.3 (6.1)	11.9 (6.0)	12.7 (6.2)	12.6 (6.2)	12.5 (6.2)	0.003
^b^VST-simple^c^	657.7 (161.6)	667.9 (172.7)	660.7 (178.0)	645.6 (121.9)	646.9 (151.8)	0.01
^b^VST-Complex^c^	2227.7 (422.9)	2267.2 (431.6)	2230.4 (462.7)	2192.3 (350.6)	2191.1 (414.3)	<0.001
NART^c^	17.9 (10.3)	18.0 (10.4)	16.2 (10.1)	18.1 (10.0)	19.7 (10.5)	<0.001
Composite score	7.7 (1.8)	7.3 (1.8)	7.9 (1.8)	7.8 (1.8)	7.8 (1.6)	<0.001
Pros.Mem % failed (*N*)	22.3 (838)	24.7 (337)	21.7 (202)	19.8 (139)	20.7 (145)	0.04

		Women				
	All	Inactive	Moderately inactive	Moderately active	Active	*P*-value
	*N* = 4744^a^	*N* = 1739	*N* = 1509	*N* = 792	*N* = 640	
**Mean (SD)**						
Age	68.1 (8.0)	70.8 (8.2)	67.2 (7.5)	66.0 (7.4)	65.3 (7.3)	<0.001
Frequencies, % (*N*)						
Retired from main occupation	78.8 (3549)	84.1 (788)	79.0 (1180)	76.8 (899)	75.7 (682)	<0.001
**Co-morbidities**						
Heart attack	2.0 (93)	3.1 (54)	1.8 (27)	0.8 (6)	0.9 (6)	<0.001
Stroke	1.4 (66)	2.1 (37)	1.3 (19)	0.9 (7)	0.5 (3)	0.01
Cancer	11.1 (528)	12.5 (217)	10.7 (161)	9.1 (72)	10.6 (68)	0.07
Diabetes	2.3 (109)	2.9 (50)	1.9 (29)	1.8 (14)	2.3 (15)	0.2
Depression	28.0 (1328)	29.0 (504)	27.4 (414)	30.2 (239)	23.6 (151)	0.03
**Alcohol (units/week)**						
0	36.0 (1638)	43.1 (731)	31.7 (465)	30.4 (234)	33.3 (208)	<0.001
≤14 Units	58.5 (2664)	52.4 (889)	62.4 (914)	62.5 (481)	60.8 (380)	
>14 Units	5.6 (253)	4.4 (75)	5.9 (86)	7.1 (55)	5.9 (37)	
Current smokers, % (*n*)	4.5 (212)	5.8 (100)	3.4 (51)	4.2 (33)	4.4 (28)	0.01
**Mean Cognitive Test Score (SD)**						
SF-EMSE	32.7 (3.0)	32.2 (3.2)	33.0 (2.8)	33.0 (3.0)	32.8 (2.7)	<0.001
HVLT	26.0 (5.5)	25.0 (5.9)	26.7 (5.3)	26.6 (5.1)	26.8 (5.0)	<0.001
PAL-FTMS	15.8 (4.2)	15.3 (4.3)	16.1 (4.2)	16.1 (4.1)	16.1 (4.2)	<0.001
PW-Accuracy	13.8 (5.9)	13.0 (6.1)	14.5 (5.8)	14.3 (5.7)	14.2 (5.8)	<0.001
^b^VST-simple^c^	668.7 (169.9)	685.6 (189.4)	661.6 (172.0)	660.1 (152.5)	651.4 (122.3)	<0.001
^b^VST-Complex^c^	2172.3 (432.5)	2208.1 (444.4)	2159.6 (469.6)	2136.1 (385.6)	2149.4 (361.4)	0.001
NART^c^	16.6 (9.5)	17.2 (9.5)	15.7 (9.4)	16.2 (9.5)	17.5 (9.4)	<0.001
Composite score	8.1 (1.8)	7.3 (1.8)	7.9 (1.8)	7.8 (1.8)	7.8 (1.6)	<0.001
Pros.Mem % failed (*N*)	15.6 (738)	19.3 (327)	12.5 (185)	16.0 (125)	14.5 (91)	<0.001

HVLT, Hopkins Verbal Learning Test; *N*, number; NART, National Adult Reading Test; PAL-FTMS, Paired Associates Learning, First Trial Memory Score; Pros.Mem, Prospective Memory SF-EMSE, Short Form Extended Mental State Exam; VST, Visual Sensitivity Test.

a Totals do not match due to missing data.

b Reaction time measured in milliseconds.

c Higher scores indicate lower performance.

Table 3 shows associations of habitual inactivity cross-sectionally and prospectively, across individual cognitive tests (assessing a range of domains) and the composite score. After controlling for age and sex, the most attenuation occurred for the bottom 10^th^ percentile after adjusting for education and social class with further adjustments for other co-variables, making little difference to the point estimates. Apart from the NART, where education and social class strengthened the association with cognition, there was little change across the models for the top 10^th^ percentile for the other tests.

**Table 3:** Association (prospective and cross-sectional) between physical inactivity and cognitive performance for eight cognitive measures separately and composite score for participants taking part in EPIC-Norfolk, 2006–2011 (including data from the pilot phase 2004–2006)

		Model 1						Model 2					Model 3			
Inactive vs active*	Ref.**	Bottom 10th percentile				Top 10th percentile		Bottom 10th percentile		Top 10th percentile			Bottom 10th percentile		Top 10th percentile	
	OR	OR	(95% CI)			OR	(95% CI)	OR	(95% CI)	OR	(95% CI)	*N*	OR	(95% CI)	OR	(95% CI)
**SF-EMSE**																
Cross-sec. (*N* = 8368)	1.00	1.20	(1.04, 1.37)			0.82	(0.71, 0.96)	1.08	(0.94, 1.24)	0.90	(0.77, 1.05)	8289	1.08	(0.94, 1.24)	0.90	(0.77, 1.05)
		*P* = 0.01				*P* = 0.01		*P* = 0.3		*P* = 0.2			*P* = 0.3		*P* = 0.2	
Prospective (*N* = 8483)	1.00	0.99	(0.85, 1.16)			1.06	(0.90, 1.25)	0.96	(0.82, 1.13)	1.08	(0.91, 1.28)	8356	0.96	(0.81, 1.12)	1.06	(0.90, 1.26)
		*P* = 0.9				*P* = 0.5		*P* = 0.6		*P* = 0.4			*P* = 0.6		*P* = 0.5	
**HVLT**																
Cross-sec. (*N* = 8028)	1.00	1.19	(1.02, 1.39)			0.83	(0.71, 0.97)	1.12	(0.95, 1.31)	0.93	(0.79, 1.09)	7954	1.10	(0.94, 1.30)	0.93	(0.79, 1.10)
		*P* = 0.03				*P* = 0.02		*P* = 0.2		*P* = 0.4			*P* = 0.2		*P* = 0·3	
Prospective (*N* = 8138)	1.00	1.09	(0.92, 1.30)			1.20	(1.02, 1.42)	1.08	(0.90, 1.29)	1.25	(1.06, 1.49)	8020	1.07	(0.90, 1.28)	1.23	(1.04, 1.47)
		*P* = 0.3				*P* = 0.03		*P* = 0.4		*P* = 0.01			*P* = 0.4		*P* = 0.02	
**PAL- FTMS**																
Cross-sec. (*N* = 7352)	1.00	1.24	(1.06, 1.45)			0.97	(0.82, 1.14)	1.16	(0.99, 1.36)	1.02	(0.86, 1.21)	7283	1.16	(0.99, 1.36)	1.01	(0.85, 1.19)
		*P* = 0.01				*P* = 0.7		*P* = 0.07		*P* = 0.8			*P* = 0·07		*P* = 0.9	
Prospective (*N* = 7461)	1.00	0.86	(0.71, 1.03)			0.96	(0.80, 1.16)	0.85	(0.70, 1.02)	0.97	(0.81, 1.18)	7352	0.84	(0.70, 1.02)	0.96	(0.79, 1.16)
		*P* = 0.09				*P* = 0.7		*P* = 0.08		*P* = 0.8			*P* = 0.07		*P* = 0.7	
**PW-Accuracy**																
Cross-sec. (*N* = 8296)	1.00	1.03	(0.89, 1.19)			0.93	(0.79, 1.10)	0.99	(0.85, 1.14)	0.96	(0.82, 1.13)	8219	0.97	(0.84, 1.13)	0.98	(0.83, 1.16)
		*P* = 0.7				*P* = 0.4		*P* = 0.9		*P* = 0.6			*P* = 0·7		*P* = 0·8	
Prospective (*N* = 8410)	1.00	0.98	(0.83, 1.15)			1.09	(0.91, 1.30)	0.99	(0.84, 1.17)	1.10	(0.92, 1.32)	8285	0.99	(0.84, 1.17)	1.14	(0.95, 1.36)
			*P* = 0.8		*P* = 0.4			*P* = 0.9		*P* = 0.3			*P* = 0.9		*P* = 0.2	
**VST-simple (reaction time, ms)**																
Cross-sec. (*N* = 7067)	1.00	1.06	(0.90, 1.25)			0.77	(0.64, 0.92)	0.99	(0.84, 1.18)	0.77	(0.64, 0.92)	6999	0.99	(0.84, 1.18)	0.76	(0.64, 0.92)
				*P* = 0.5		*P* = 0.004		*P* = 0.9		*P* = 0.004			*P* = 0.9		*P* = 0.004	
Prospective (*N* = 7171)	1.00	1.06	(0.88, 1.28)			1.00	(0.82, 1.21)	1.05	(0.87, 1.27)	0.99	(0.81, 1.21)	7062	1.04	(0.86, 1.26)	0.99	(0.81, 1.20)
				*P* = 0.5		*P* = 0.9		*P* = 0.6		*P* = 0.9			*P* = 0·7		*P* = 0.9	


		Model 1				Model 2					Model 3			
Inactive vs active*	REF**OR	Bottom 10th percentile		Top 10th percentile		Bottom 10th percentile		Top 10th percentile			Bottom 10th percentile		Top 10th percentile	
**VST-Complex (reaction time, ms)**														
Cross-sec. (*N* = 7067)	1.00	1·25	(1.06, 1.48)	0.94	(0.79, 1.11)	1.22	(1.03, 1.44)	0.93	(0.78, 1·10)	6999	1.20	(1.02, 1.42)	0.93	(0.78, 1.11)
		*P* = 0.01		*P* = 0.5		*P* = 0.02		*P* = 0.4			*P* = 0.03		*P* = 0.4	
Prospective (*N* = 7171)	1.00	1·22	(1.02, 1.46)	1.02	(0.84, 1.23)	1.23	(1.02, 1.45)	1.01	(0.84, 1.23)	7062	1.20	(0.99, 1.44)	1.01	(0.83, 1.22)
		*P* = 0.03		*P* = 0.9		*P* = 0.03		*P* = 0.9			*P* = 0.06		*P* = 1.00	
**NART**														
Cross-sec. (*N* = 8002)	1.00	1.03	(0.88, 1.20)	0.84	(0.72, 0.98)	0.89	(0.76, 1.05)	1.06	(0.89, 1.25)	7922	0.89	(0.75, 1.05)	1.07	(0.90, 1.26)
		*P* = 0.7		*P* = 0.03		*P* = 0.2		*P* = 0.5			*P* = 0.2		*P* = 0.5	
Prospective (*N* = 8112)	1.00	0.87	(0.72, 1.04)	1.18	(1.00, 1.40)	0.88	(0.73, 1.07)	1.38	(1.15, 1.66)	8000	0.88	(0.72, 1.06)	1.37	(1.14, 1.65)
		*P* = 0.1		*P* = 0.05		*P* = 0.2		*P* = 0.001			*P* = 0.2		*P* = 0.001	
**Composite score**														
Cross-sec. (*N* = 6061)	1.00	1.28	(1.11, 1.47)	0.83	(0.70, 0.97)	1.18	(1.02, 1.37)	0.91	(0.77, 1.07)	6002	1.17	(1.01, 1.36)	0.91	(0.77, 1.07)
		*P* = 0.001		*P* = 0.02		*P* = 0.03		*P* = 0.3			*P* = 0.04		*P* = 0.3	
Prospective (*N* = 6152)	1.00	0.87	(0.74, 1.02)	1.13	(0.95,1.34)	0.86	(0.72, 1.02)	1.18	(0.98, 1.41)	6057	0.85	(0.71, 1.01)	1.15	(0.96, 1.38)
		*P* = 0.1		*P* = 0.2		*P* = 0.08		*P* = 0.08			*P* = 0.07		*P* = 0.1	
	REF***	Fail				Fail					Fail			
Pros. Mem**		OR	(95% CI)			OR	(95% CI)				OR	(95% CI)		
Cross-sec. (*N* = 8290)	1.00	1.03	(0.91, 1.16)			1.03	(0.91, 1.16)			8213	0.98	(0.87, 1.11)		
		*P* = 0·6				*P* = 0.6					*P* = 0.8			
Prospective (*N* = 8403)	1.00	0.81	(0.70, 0.93)			0.8	(0.69, 0.92)			8278	0.79	(0.69, 0.91)		
		*P* = 0.003				*P* = 0.001					*P* = 0.001			

Model 1: Adjusted age per 5-year increase (at time of cognitive testing) and sex. Model 2: Adjusted age per 5-year increase (at time of cognitive testing, or 3HC), sex, education (at three levels: 1/no qualifications, 2/O and A level, and 3/degree and above), social class (at two levels: manual and non-manual). Model 3: Adjusted age per 5-year increase (at time of cognitive testing at 3HC), sex, education (at three levels: 1/no qualifications, 2/O and A level, and 3/degree and above from baseline), social class (at two levels: manual and non-manual from baseline), prevalent disease (at baseline and at 3HC) and smoking (at two levels: smokers vs non-smokers, at baseline and at 3HC).

Reference categories are active*, 11th–89th** percentile and success*** group, respectively.

A, Advanced; Cross-sec., cross-sectional analysis; HVLT, Hopkins Verbal Learning Test; ms, milliseconds; *N*, number; NART, National Adult Reading Test; O, Ordinary; PAL-FTMS, Paired Associates Learning, First Trial Memory Score; Pros.Mem, Prospective Memory; SF-EMSE, Short Form Extended Mental State Exam; VST, Visual Sensitivity Test.

For most tests, there was little or no relationship between habitual inactivity and cognition. However, being inactive was positively associated with poor performance for VST-complex in both cross-sectional and possibly also in the prospective analyses {odds ratio [OR] = 1.20, [95% confidence interval (CI) 1.02, 1.42], *P* = 0.03; OR = 1.20 [95% CI 0.99, 1.44], *P* = 0.06}, respectively. Inactive participants were also less likely to perform poorly in the prospective analysis for the prospective memory task [OR = 0.79 (95% CI 0.69, 0.91, *P* = 0.001)].

Physical inactivity increased the likelihood of high performance for the tests: Hopkins Verbal Learning Test (HVLT) [OR = 1.23 (95% CI 1.04, 1.47), *P* = 0.02] and NART [OR = 1.37 (95% CI 1.14, 1.65), *P* = 0.001], but only for the prospective analyses. For VST-Simple, those inactive at 3HC were less likely to be high performers. No associations were observed for poor performance. For the composite score, inactivity was associated with increased risk of poor performance cross-sectionally and a possible decreased risk prospectively. For practicality, only the composite score was used to examine the relationship with work and leisure separately. Table 4 shows these associations in men and women with further adjustment for total habitual activity (Model 4).

**Table 4:** Association between physical inactivity (leisure and occupation time separately) with cognition (using composite score only) in the EPIC-Norfolk Cohort (including pilot data) for men and women combined as well as separately

		Model 3							Model 4**						
Inactive vs active*	Ref.**	Bottom 10th percentile			Top 10th percentile			Ref.**	Bottom 10th percentile			Top 10th percentile			
	OR	OR	(95% CI)	*P*-value	OR	(95% CI)	*P*-value	OR	OR	(95% CI)	*P*-value		OR	(95% CI)	*P*-value
**Leisure activity only**															
**(All)**															
Cross-sec. (*N* = 6002)	1.00	1.24	(1.07, 1.43)	0.004	0.95	(0.82, 1.12)	0.6	1.00	1.27	(1.00, 1.61)	0.05	1.07		(0.83, 1.36)	0.6
Prospective (*N* = 6057)	1.00	1.01	(0.88, 1.17)	0.9	0.96	(0.82, 1.12)	0.6	1.00	1.14	(0.96, 1.36)	0.1	0.81		(0.66, 1.00)	0.05
**Men**															
Cross-sec. (*N* = 2693)	1.00	1.34	(1.09, 1.64)	0.01	1.09	(0.85, 1.40)	0.5	1.00	1.35	(1.00, 1.82)	0.05	1.31		(0.91, 1.88)	0.2
Prospective (*N* = 2725)	1.00	0.97	(0.80, 1.18)	0.8	0.79	(0.61, 1.01)	0.06	1.00	1.17	(0.93, 1.48)	0.2	0.60		(0.42, 0.87)	0.01
**Women**															
Cross-sec. (*N* = 3309)	1.00	1.13	(0.92, 1.39)	0.3	0.87	(0.72, 1.07)	0.2	1.00	1.14	(0.77, 1.71)	0.5	0.91		(0.65, 1.27)	0.6
Prospective (*N* = 3332)	1.00	1.04	(0.84, 1.28)	0.7	1.08	(0.89, 1.32)	0.5	1.00	1.05	(0.81, 1.37)	0.7	0.95		(0.73, 1.23)	0.7
**Work activity only (all)**															
Cross-sec. (*N* = 2756)	1.00	0.65	(0.50, 0.85)	0.002	1.21	(0.99, 1.48)	0.06	1.00	0.65	(0.45, 0.93)	0.02	1.19		(0.95, 1.50)	0.1
Prospective (*N* = 5020)	1.00	0.66	(0.55, 0.79)	<0.001	1.21	(1.03, 1.43)	0.02	1.00	0.68	(0.54, 0.86)	0.001	1.16		(0.96, 1.40)	0.1
**Men**															
Cross-sec. (*N* = 1388)	1.00	0.75	(0.52, 1.06)	0.1	1.31	(0.95, 1.80)	0.1	1.00	0.70	(0.43, 1.13)	0.1	1.39		(0.97, 2.00)	0.08
Prospective (*N* = 2275)	1.00	0.70	(0.54, 0.91)	0.01	1.21	(0.92, 1.61)	0.2	1.00	0.77	(0.56, 1.07)	0.1	1.27		(0.93, 1.73)	0.1
**Women**															
Cross-Sec. (*N* = 1368)	1.00	0.53	(0.34, 0.82)	0.004	1.18	(0.91, 1.53)	0.2	1.00	0.59	(0.33, 1.04)	0.07	1.10		(0.81, 1.50)	0.5
Prospective (*N* = 2745)	1.00	0.63	(0.47, 0.83)	0.001	1.20	(0.98, 1.47)	0.08	1.00	0.59	(0.41, 0.84)	0.004	1.08		(0.85, 1.37)	0.5

Model 3: Adjusted age per 5-year increase (at time of cognitive testing, or 3HC), sex, education (at three levels: 1/no qualifications, 2/O and A level, and 3/degree and above from baseline), social class (at two levels: manual and non-manual from baseline), prevalent disease (at baseline and time of cognitive testing, 3HC) and smoking (at two levels: smokers vs non-smokers), all covariates measures entered from baseline and at 3HC separately.

Reference categories are active* and 11th–89th** percentile group, respectively.

Cross-sect: cross-sectional analysis; *N*, number; OR, odds ratio; **Model 4: as in Model 3 with further adjustment for total physical activity as categorical variables.

Leisure inactivity seemed to increase risk of poor performance, cross-sectionally [OR = 1.27 (95% CI 1.00, 1.61), *P* = 0.05]. For high performance, no relationship was observed cross-sectionally, but a possible inverse relationship was observed for the prospective analyses. There seemed to be some indication of an increased risk with poor performance for inactivity in men cross-sectionally and decrease in being in the top performance prospectively. No clear relationship was observed for women.

In contrast to leisure time, inactivity during work was associated with lower risk of poor performance with little difference observed in the cross-sectional and prospective analyses. In relation to high performance, the relationship was stronger for men, with a possible increased likelihood of high performance observed for inactive working men, but not for inactive working women (Table 4).

**Figure 2:**
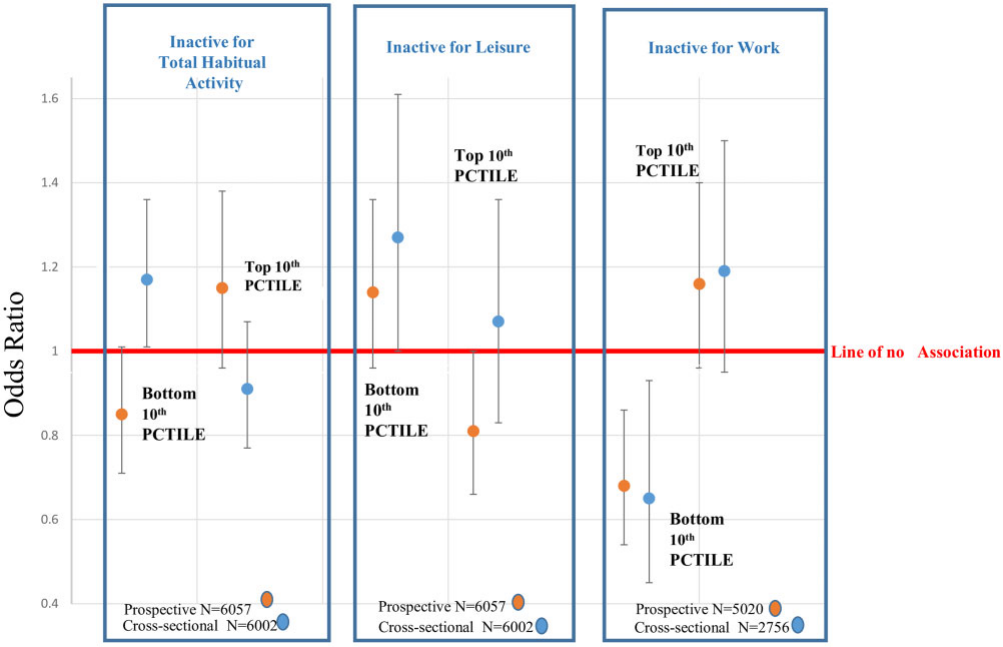
Diagrammatic representation of the relationship between inactivity and cognitive performance for total habitual, as well as leisure and work-time activity separately, at the two time points (men and women combined). The relationship between inactivity and cognition is clearer with the separation of work and leisure-time activity. Inactive at leisure is associated with increased risk of poor cognition, whereas inactive at work is associated with a lower risk of poor cognition.

**Figure 3:**
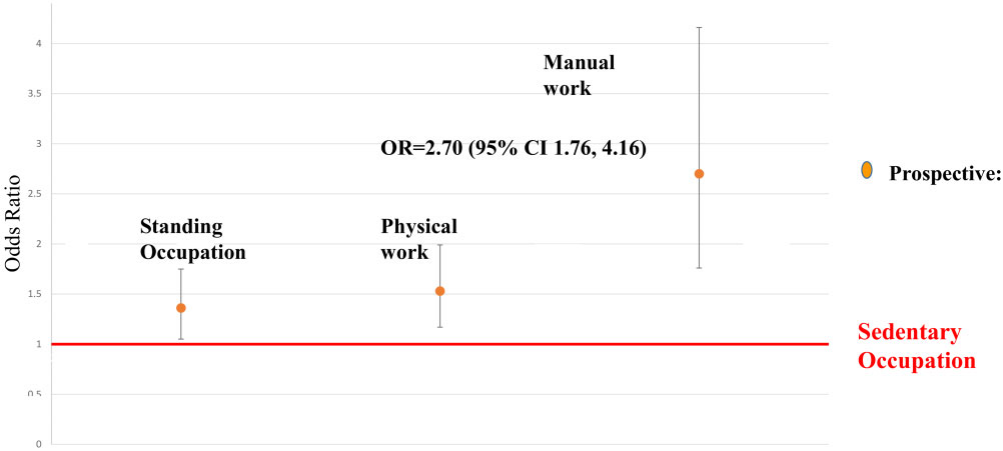
Increasing work-related activity has a greater risk of poor performance for those in manual work, having an almost three times higher risk of poor performance than those with a sedentary job. CI, confidence interval; OR, odds ratio.

Figure 2 is a visual representation of the relationship between inactivity and cognitive performance for total habitual, as well as leisure and work-time activity separately, at the two time points (men and women combined). Figure 3 shows that increased work-related physical activity (as reported at baseline) was associated with increased risk of poor performance, with manual workers having a greater risk of poor performance than those with physically inactive occupations [OR = 2.70 (95% CI 1.76, 4.16), *P* < 0.001].

No significant interaction was observed with education and work-related activity either cross-sectionally (bottom and top 10th percentiles, *P* = 0.4 and *P* = 0.9, respectively) or prospectively (bottom and top 10th percentiles, *P* = 0.6 and *P* = 0.5, respectively). However, those with no qualifications were less likely to be inactive at work and more likely to be inactive at leisure (Supplementary Table 2, available as Supplementary data at *IJE* online). On stratification (Supplementary Table 3, available as Supplementary data at *IJE* online), results indicate that being inactive at work reduced the risk of poor performance for those both with and without qualifications. Furthermore, those remaining in an inactive job at the time of cognitive testing increased their probability of being in the top 10th percentile [OR= 1.29 (95% CI, 1.02, 1.62), *P* = 0.03]. The risk for poor performance increased for those inactive for leisure, particularly for those ‘with qualifications’ [OR= 1.35 (95% CI, 1.00, 1.81), *P* = 0.05].

In a sensitivity analysis, imputing poor performance for missing data made little difference to the OR (Supplementary Table 4, available as Supplementary data at *IJE* online), suggesting that the missing data do not reduce the representativeness of the sample. The analyses based on approximate quartiles of cognitive scores (Supplementary Tables 5 and 6, available as Supplementary data at *IJE* online) also showed a threshold association with physical inactivity. Thus, changing the grouping made little difference to the overall findings. Given that this study is in apparently healthy older adults, the more stringent cut-off as used in the main analysis is more appropriate.

## Discussion

This analysis of cognition in a mid-life population-derived cohort reveals a differential in association between cognition and inactivity during work and leisure. Work-related physical activity does not protect against poor cognitive performance. Those reporting an inactive occupation had a lower future risk of poor cognition and were more likely to have higher performance in cognitive tests in later life—a finding most obvious in men.

One limitation of this study is of healthy-volunteer bias and lower representation of the poor-cognition group. Nevertheless, our study still includes a wide range of individuals in terms of social class, education, age and cognitive ability, and both men and women, as in the general population.^22^ Another limitation is the inability to control for other early-life indicators such as prior intelligence, family social-economic status and parental education, which are known determinants of cognitive function,^23^ but were not available in this cohort.

The use of a self-report measure of physical activity may be criticized as prone to recall biases and not as accurate as an objective measure. This index was derived based on self-reported classification of the level of certain leisure activities and the type of work participants typically did. We did not quantify the level of inactivity. However, this index has been validated and shown to predict cardiovascular disease and mortality.^24^ Its greatest advantage is its simplicity and usability in different settings. Finally, due to the nature of the design of the study as an observational study, adjusting for the unequal distribution of the potential confounders is always limited and there may be residual confounding.

The principal strength of this study is the in-depth exploration of particular types of physical activity and the relationship with cognitive function. We observed differential associations between work and leisure-time inactivity, and show how the varying distribution of these activities in populations, or, as in this study, at different time points, may influence associations observed, This has not been explored previously. We also report differences across socio-demographic factors. Other cohorts have been limited in their breadth of socio-demographic factors, with either insufficient^5^ or over-representation of more educated, ‘white-collar’ or more affluent individuals.^6^^,^^25^

Although no particular pattern was observed by cognitive test (domain), the relationship did vary by test. The strongest relationships between habitual (total) inactivity and high cognitive performance were observed for NART and HVLT. NART is a test of accumulated knowledge, which is a recognized benefit of education.^26^ NART has previously shown to be strongly associated with education and social class, followed by HVLT, a verbal memory test that involves recognition of words.^22^ Both tests assess domains subject to the influence of education. Inactivity was only associated with increased poor performance in the VST-complex measure. Reaction-time tests assessing more basic processes than the other higher-order complex cognitive tasks^27^ are less affected by socio-economic and educational differences.^28^ Inconsistencies observed previously could be explained to some degree by the choice of the cognitive-assessment tool.

Physical inactivity during leisure time was more strongly associated with poor performance for men in the cross-sectional analysis and the inverse relationship between inactivity during work was stronger in women. However, in terms of high performance, occupational inactivity was stronger for men only. The reasons for this may well reflect the use of a partner’s occupation for classifying women’s social class. A woman classified by her partner’s manual social class may not necessarily have the same physical-activity patterns as her partner, although this could be further evidence of confounding by social class.

Unlike others studies, we observed little evidence of reverse causation.^4–6^^,^^9^ The differential relationship between inactivity and cognitive function was only revealed by stratifying the components of the physical-activity index into work and leisure-time activity—something not done previously. Studies reporting reverse causation as a potential bias have used moderate and severe cognitive impairment including dementia^4–6^^,^^9^ as the outcome measure, with less interest in the milder cognitive dysfunction. Cognitive impairment and dementia have a long prodromal period resulting in individuals having reduced physical activity and more likely to be lost to follow-up.

In our healthier population with no overt cognitive impairment, there was loss to follow-up and reverse causation could not be entirely ruled out. However, this is unlikely to be to the same degree as those observed for studies using clinical dementia-based outcomes, particularly for the cross-sectional analyses. We believe it is important to examine the more subtle dysfunction in cognition that affects the vast majority of the ageing population and has a differential impact on the quality of life and daily functioning.

Our results are consistent with other studies showing a positive relationship with poor cognition and leisure-time inactivity^9^^,^^29^^,^^30^ and work-related activity,^5^ with increasing physical work of manual workers having a greater risk of poor performance. We also found a physically inactive job (typically a desk job) reduces the risk of poor cognition irrespective of education. This may be because a desk job is likely to be more cognitively demanding than a manual occupation and strengthens our findings of confounding by education, occupation and social class. The observations for leisure activity also provide further evidence of confounding by differential leisure-time pursuits according to education and social class.

We address a number of issues raised in previous reports calling for stronger evidence on physical activity for preventing cognitive decline, impairment and dementia.^7^^,^^31^ Despite adjusting for a range of cofactors including education, social class and health, other studies have not been able to adequately address the issue of residual confounding. We conclude that the relationship between inactivity and cognition is complex and risk factors are not independent of each other. Though promoting physical activity can do no harm, policy makers must be transparent about the evidence and the limitations of confounding before embarking on any health-promotion strategies so as not to lose public support by giving mixed messages.

Further studies are needed, in particular, on inequalities across socio-economic groups and the impact of lower education, poor-quality work (shortage of beneficial physical and mental stimulation), particularly for manual labour, and the lack of opportunity and space to be physically active for leisure. All these are key drivers that provide fewer opportunities to build cognitive reserve to protect for cognitive impairment and dementia in later life.^13^ Future studies should use methods that clearly discriminate between work and leisure, and be more specific on the nature of inactivity with good representation across socio-economic groups.

## Author contributions

Study concepts and design: S.A.H., R.L., K.T.K., C.B., N.W.; Participant recruitment and data collection: S.A.H., R.L., K.T.K., N.W.; Analysis and interpretation of data: S.A.H., C.B. and K.T.K. Manuscript: S.A.H. drafted and wrote the manuscript with review and contributions from co-authors. All authors have read and approved the manuscript.

## Funding

This work was supported by the Medical Research Council, UK (MRC) http://www.mrc.ac.uk/ (Ref: MR/N003284/1, MC-UU_12015/1 to N.W.); Cancer Research UK, http://www.cancerresearchuk.org/ (CRUK, Ref: C864/A8257) and NIHR, https://www.nihr.ac.uk (Ref: NF-SI-0616–10090 to C.B.). The clinic for EPIC- orfolk 3HC was funded by Research into Ageing, now known as Age UK, http://www.ageuk.org.uk/ (Grant Ref: 262). The pilot phase was supported by MRC (Ref: G9502233) and CRUK (Ref: C864/A2883).

## Supplementary Material

dyaa067_Supplementary_Data
