# Supplementary material for: Cross-sectional and prospective relationship between occupational and leisure-time inactivity and cognitive function in an ageing population: the European Prospective Investigation into Cancer and Nutrition in Norfolk (EPIC-Norfolk) study
Source: Int J Epidemiol. 2020 Jul 6;49(4):1338–52. doi: 10.1093/ije/dyaa067 (PMC7660150; doi:10.1093/ije/dyaa067)
Supplement: dyaa067_Supplementary_Data [file dyaa067_supplementary_data.docx]

**Supplementary Table S1: Characteristics of individuals by those invited, attended 3HC with a cognitive test measure compared to those who either did not attend the EPIC-Norfolk 3 (or the pilot) or had no cognitive test measure.**

|  | **Total Invited (N=18,382)** | |  |
| --- | --- | --- | --- |
|  | Attenders, with cog score(N=8585) | Non-attenders (N=9797)* | P-Value |
| **Characteristics at Baseline** |  |  |  |
| **Mean (SD)** |  |  |  |
| **Age** | 55·7 (7·8) | 58·9 (9·3) | <0·001 |
|  |  |  |  |
| **Frequencies, % (N)** |  |  |  |
| **% men** | 44·7 (3841) | 42·0 (4117) | <0·001 |
| **Level of education** |  |  |  |
| No Qualification | 26·2 (2251) | 45·1 (4414) | <0·001 |
| O or A level | 56·1 (4521) | 46·2 (4521) |  |
| Graduate Level or above | 17·6 (1513) | 8·7 (856) |  |
| **Social Class** |  |  |  |
| Professional | 8·8 (748) | 4·9 (468) | <0·001 |
| Managerial | 41·1 (3498) | 30·1 (2867) |  |
| Skilled Non-Manual | 16·0 (1364) | 17·1 (1635) |  |
| Skilled Manual | 20·6 (1748) | 26·8 (2555) |  |
| Semi-Skilled | 11·2 (950) | 16·3 (1550) |  |
| Non-Skilled | 2·3 (197) | 4·8 (459) |  |
|  |  |  |  |
| **Alcohol (Units/week)** |  |  |  |
| 0 | 8·9(763) | 15·5 (2263) | <0·001 |
| ≤ 14 Units | 75·2 (6426) | 71·6 (6912) |  |
| > 14 Units | 15·8 (1353) | 12·8 (1239) |  |
| **Smoking Status** |  |  |  |
| Never | 52·2 (4463) | 45·0(4365) | <0·001 |
| Former | 38·9 (3329) | 41·0 (3971) |  |
| Current | 8·9 (760) | 1. (1361) |  |
| **Co-morbidities** |  |  |  |
| Heart attack | 1·5 (128) | 2·6 (251) | <0·001 |
| Stroke | 0·6 (50) | 1·0 (99) | 0·001 |
| Cancer | 4·4 (374) | 4·4 (426) | 1·00 |
| Diabetes | 1·0 (85) | 2·2 (218) | <0·001 |
| Depression | 14·6 (1250) | 14·3 (1398) | 0·6 |
|  |  |  |  |

P- Value Using Anova or chi sq

(* includes 38 who attended but had no cognition score). Abbreviations: A, Advanced; N, Number; O, Ordinary; SD, standard deviation

**Supplementary Table S2: Distribution of inactivity during leisure and work by education level (cross-sectional and prospective analysis)**

|  | **Frequencies, % (N)** | |  |
| --- | --- | --- | --- |
|  | **No Qualifications** | **Any Qualifications** | **P-Value** |
| **Cross-sectional** |  |  |  |
| Inactive at work (N=1675) | 25·7 (183) | 48·3 (1492) | <0·001 |
| Inactive at leisure (N=4098) | 58·0 (1282) | 45·0 (2816) | <0·001 |
| **Prospective** |  |  |  |
| Inactive at work at baseline (N=2742) | 22·6 (372) | 44·3 (2370) | <0·001 |
| Inactive at leisure (N=3661) | 50·6 (1138) | 39·8 (2523) | <0·001 |

**Testing for Interaction**

The dichotomised activity index for occupation only and the dichotomised variable for education (Qualifications vs No Qualifications were combined to create a work activity-education variable, this was then entered as a separate variable into model 4.

Due to the strong influence of education on cognition, data were stratified by education groups (‘No Qualifications’ and ‘With Qualifications’). Adjusted odd ratios in each group for both work and leisure are presented in Table S3 below. Stratification allows further exploration of interaction and potential confounding.

**Supplementary Table S3: Association between physical activity (leisure and occupational separately) and cognitive performance (using composite score) in the EPIC-Norfolk 3 Cohort (2006-2010), including pilot data (2004-2006) stratified by education**.

|  |  | **No Qualifications** | | | | | |  | **With Qualifications** | | | | | |
| --- | --- | --- | --- | --- | --- | --- | --- | --- | --- | --- | --- | --- | --- | --- |
| **Inactive vs Active*** | **Inactive, % (N)** | **Bottom 10th percentile** | | | **Top 10th percentile** | | | **Inactive, % (N)** | **Bottom 10th percentile** | | | **Top 10th percentile** | | |
|  |  | **OR** | **(95% CI)** | **P-Value** | **OR** | **(95% CI)** | **P-Value** |  | **OR** | **(95% CI)** | **P-Value** | **OR** | **(95% CI)** | **P-Value** |
| **Leisure Activity** |  |  |  |  |  |  |  |  |  |  |  |  |  |  |
| Cross-sectional (N=6002) | 56·5 (831) | 1·17 | (0·79, 1·73) | 0·4 | 1·30 | (0·61, 2·76) | 0·5 | 43·9 (1988) | 1·35 | (1·00, 1·81) | 0·05 | 0·94 | (0·73, 1·22) | 0·7 |
| Prospective (N=6057 ) | 49·8 (743) | 1·17 | (0·79, 1·73) | 0·4 | 1·30 | (0·61, 2·76) | 0·5 | 40·4 (1842) | 1·35 | (1·00, 1·81) | 0·05 | 0·94 | (0·73, 1·22) | 0·7 |
| **Work Activity** |  |  |  |  |  |  |  |  |  |  |  |  |  |  |
| Cross-sectional (N=2756) | 16·7 (81) | 0·57 | (0·22, 1·47) | 0·2 | 1·22 | (0·37, 3·99) | 0·7 | 48·6 (1104) | 0·64 | (0·43, 0·94) | 0·02 | 1·29 | (1·02, 1·62) | 0·03 |
| Prospective (N=5020 ) | 23·5 (263) | 0·57 | (0·35, 0·93) | 0·02 | 1·00 | (0·45, 2·18) | 1·00 | 44·8 (1747) | 0·71 | (0·54, 0·94) | 0·02 | 1·17 | (0·97, 1·42) | 0·1 |

*Active=reference category ^Ɨ^ 11-89 percentile =reference category

**Supplementary Table S4:** **Association between physical activity (leisure and occupation time separately) with cognition (using composite score only) in the EPIC-Norfolk Cohort by including those with missing data as being in poor performance group for each test (Sensitivity Analysis I).**

|  | Model 3 | | | | | | | Model 4*** | | | | | | |
| --- | --- | --- | --- | --- | --- | --- | --- | --- | --- | --- | --- | --- | --- | --- |
| **Inactive vs Active*** | **REF**** | **Bottom 10th percentile** | | | **Top 10th percentile** | | | **REF **** | **Bottom 10th percentile** | | | **Top 10th percentile** | | |
|  | **OR** | **OR** | **(95% CI)** | **P-Value** | **OR** | **(95% CI)** | **P-Value** | **OR** | **OR** | **(95% CI)** | **P-Value** | **OR** | **(95% CI)** | **P-Value** |
| **Leisure Activity Only** |  |  |  |  |  |  |  |  |  |  |  |  |  |  |
| Cross-Sec (N=8386) | 1·00 | 1·17 | (1·01, 1·36) | 0·03 | 0·92 | (0·79, 1·06) | 0·2 | 1·00 | 1·23 | (0·97, 1·58) | 0·1 | 0·95 | (0·75, 1·20) | 0·6 |
| Prospective (N= 8457) | 1·00 | 1·05 | (0·91, 1·21) | 0·5 | 1·03 | (0·89, 1·20) | 0·6 | 1·00 | 1·06 | (0·89, 1·27) | 0·5 | 0·87 | (0·71, 1·06) | 0·2 |
| **Work Activity Only** |  |  |  |  |  |  |  |  |  |  |  |  |  |  |
| Cross-Sec (N=3769) | 1·00 | 0·74 | (0·56, 0·97) | 0·03 | 1·29 | (1·07, 1·56) | 0·01 | 1·00 | 0·64 | (0·43, 0·94) | 0·02 | 1·28 | (1·03, 1·59) | 0·02 |
| Prospective (N= 6892) | 1·00 | 0·72 | (0·60, 0·88) | 0·001 | 1·26 | (1·08, 1·47) | 0·003 | 1·00 | 0·66 | (0·51, 0·85) | 0·001 | 1·18 | (0·99, 1·40) | 0·07 |
| **Total activity** |  |  |  |  |  |  |  |  |  |  |  |  |  |  |
| Cross-Sec (N=8386) | 1·00 | 1·11 | (0·96, 1·29) | 0·1 | 0·91 | (0·78, 1·07) | 0·3 |  |  |  |  |  |  |  |
| Prospective (N= 8457) | 1·00 | 1·02 | (0·87, 1·21) | 0·7 | 1·23 | (1·03, 1·46) | 0·02 |  |  |  |  |  |  |  |

Model 3: Adjusted age per 5 years increase (at time of cognitive testing, or 3HC) , sex education (at 3 levels, 1/no qualifications, 2/O and A level and 3/degree and above from baseline) social class (at two levels, manual and non-manual from baseline) prevalent disease (at baseline and time of cog testing, 3HC) and smoking (at two level, smokers vs non-smokers, all co-variates measures entered from baseline and at 3HC separately

(Reference categories are active* and 11^th^-89^th^** percentile group respectively)

***Model 4: As in model 3 with further adjustment for total physical activity as categorical variable

**Supplementary Table S5:Sensitivity Analysis II showing age and sex adjusted association (prospective and cross-sectional) between physical inactivity and cognitive performance by group (of approximate quartile) for each of the eight cognitive tests and composite score for participants taking part in EPIC-Norfolk, 2006-2011 (including Data from the Pilot Phase 2004-2006).**

|  |  | **Model 1** | | |
| --- | --- | --- | --- | --- |
|  |  | **OR** | **(95% CI)** | **P-Value** |
|  | Freq, N |  |  |  |
| **Cross-sectional** |  |  |  |  |
| SF-EMSE | **8368** |  |  |  |
| G1 | 2261 | 1·44 | (1·21, 1·70) | <0·001 |
| G2 | 2252 | 1·18 | (1·00, 1·40) | 0·05 |
| G3 | 2863 | 1·16 | (0·99, 1·37) | 0·07 |
| G4 | 992 | 1·00 |  |  |
| **Prospective** |  |  |  |  |
| SF-EMSE | **8483** |  |  |  |
| G1 | 2305 | 0·94 | (0·78, 1·14) | 0·6 |
| G2 | 2276 | 0·91 | (0·76, 1·10) | 0·3 |
| G3 | 2898 | 0·97 | (0·81, 1·16) | 0·7 |
| G4 | 1004 | 1·00 |  |  |
| **Cross-sectional** |  |  |  |  |
| HVLT | 8028 |  |  |  |
| G1 | 1998 | 1·35 | (1·17, 1·56) | <0·001 |
| G2 | 2482 | 1·21 | (1·05, 1·38) | 0·01 |
| G3 | 1621 | 1·09 | (0·94, 1·26) | 0·3 |
| G4 | 1927 | 1·00 |  |  |
| **Prospective** |  |  |  |  |
| HVLT | **8138** |  |  |  |
| G1 | 2036 | 0·91 | (0·77, 1·07) | 0·2 |
| G2 | 2514 | 0·87 | (0·75, 1·01) | 0·07 |
| G3 | 1640 | 0·88 | (0·75, 1·04) | 0·1 |
| G4 | 1948 | 1·00 |  |  |
| **Cross-sectional** |  |  |  |  |
| FTMS | **7352** |  |  |  |
| G1 | 2030 | 1·18 | (1·00, 1·38) | 0·05 |
| G2 | 2067 | 1·11 | (0··95, 1·29) | 0·2 |
| G3 | 1984 | 1·05 | (0·90, 1·23) | 0·6 |
| G4 | 1271 | 1·00 |  |  |
| **Prospective** |  |  |  |  |
| FTMS | **7461** |  |  |  |
| G1 | 2074 | 0·87 | (0·72, 1·04) | 0·1 |
| G2 | 2093 | 0·93 | (0·83, 1·18) | 0·9 |
| G3 | 2012 | 1·03 | (0·87, 1·23) | 0·7 |
| G4 | 1282 | 1·00 |  |  |
| **Cross-sectional** |  |  |  |  |
| PW_Acc | **8296** |  |  |  |
| G1 | 2071 | 1·05 | (0·92, 1·21) | 0·5 |
| G2 | 2198 | 1·02 | (0·89, 1·17) | 0·8 |
| G3 | 2131 | 1·02 | (0·89, 1·17) | 0·8 |
| G4 | 1896 | 1·00 |  |  |
| **Prospective** |  |  |  |  |
| PW_Acc | **8410** |  |  |  |
| G1 | 2105 | 0·92 | (0·78, 1·08) | 0·3 |
| G2 | 2229 | 1·01 | (0·87, 1·18) | 0·9 |
| G3 | 2154 | 1·05 | (0·90, 1·22) | 0·6 |
| G4 | 1922 | 1·00 |  |  |

**Supplementary Table S5: Continued**

|  | **Model 1** | | | |
| --- | --- | --- | --- | --- |
|  |  | **OR** | **(95% CI)** | **P-Value** |
|  | Freq, N |  |  |  |
| **Cross-sectional** |  |  |  |  |
| VST-Simple | **7067** |  |  |  |
| G1 | 1765 | 1·20 | (1·04, 1·39) | 0·01 |
| G2 | 1768 | 1·09 | (0·94, 1·26) | 0·3 |
| G3 | 1776 | 1·13 | (0·98, 1·30) | 0·1 |
| G4 | 1758 | 1·00 |  |  |
| **Prospective** |  |  |  |  |
| VST-Simple | **7171** |  |  |  |
| G1 | 1790 | 1·14 | (0·97, 1·34) | 0·1 |
| G2 | 1795 | 0·99 | (0·84, 1·16) | 0·9 |
| G3 | 1795 | 1·04 | (0·88, 1·22) | 0·6 |
| G4 | 1791 | 1·00 |  |  |
| **Cross-sectional** |  |  |  |  |
| VST-Complex | **7067** |  |  |  |
| G1 | 1767 | 1·13 | (0·98, 1·31) | 0·1 |
| G2 | 1768 | 1·06 | (0·92, 1·22) | 0·5 |
| G3 | 1768 | 0·95 | (0·83, 1·10) | 0·5 |
| G4 | 1764 | 1·00 |  |  |
| **Prospective** |  |  |  |  |
| VST-Complex | **7171** |  |  |  |
| G1 | 1792 | 1·14 | (0·97, 1·34) | 0·1 |
| G2 | 1793 | 0·99 | (0·84, 1·16) | 0·9 |
| G3 | 1794 | 1·07 | (0·91, 1·25) | 0·4 |
| G4 | 1792 | 1·00 |  |  |
| **Cross-sectional** |  |  |  |  |
| NART | **8002** |  |  |  |
| G1 | 1803 | 1·10 | (0·96, 1·26) | 0·2 |
| G2 | 2183 | 1·24 | (1·09, 1·41) | 0·001 |
| G3 | 1973 | 1·10 | (0·96, 1·26) | 0·2 |
| G4 | 2043 | 1·00 |  |  |
| **Prospective** |  |  |  |  |
| NART | **8112** |  |  |  |
| G1 | 1835 | 0·77 | (0·66, 1·90) | 0·001 |
| G2 | 2219 | 0·82 | (0·71, 0·95) | 0·01 |
| G3 | 2005 | 0·86 | (0·74, 0·99) | 0·04 |
| G4 | 2053 | 1·00 |  |  |
| **Cross-sectional** |  |  |  |  |
| Composite Score* | **6061** |  |  |  |
| G1 | 1782 | 1·37 | (1·17, 1·61) | <0·001 |
| G2 | 1356 | 1·12 | (0·94, 1·32) | 0·2 |
| G3 | 1461 | 1·12 | (0·95, 1·31) | 0·2 |
| G4 | 1462 | 1·00 |  |  |
| **Prospective** |  |  |  |  |
| Composite Score* | **6152** |  |  |  |
| G1 | 1820 | 0·84 | (0·70, 1·01) | 0·07 |
| G2 | 1374 | 0·93 | (0·77, 1·12) | 0·4 |
| G3 | 1481 | 1·05 | (0·88, 1·26) | 0·6 |
| G4 | 1477 | 1·00 |  |  |

*See Appendix 3 below for details on how the composite score for the above approximate quartile group was created.

**Supplementary Table S6: Sensitivity Analysis showing association between physical inactivity (leisure and occupation time separately) with cognition using groups of approximate quartiles (for composite score only) in the EPIC-Norfolk Cohort (including pilot data).**

| **Inactive or sedentary vs Active (Comparison group)** | **Model 3** | | | **Model 4** | | |
| --- | --- | --- | --- | --- | --- | --- |
|  | **OR** | **(95% CI)** | **P-Value** | **OR** | **(95% CI)** | **P-Value** |
| **Leisure Activity Only** |  |  |  |  |  |  |
| **(ALL- men and women combined)** |  |  |  |  |  |  |
| **Cross-sectional (N=6002)** |  |  |  |  |  |  |
| G1 (N=1758) | 1·15 | (0·98, 1·36) | 0·09 | 1·08 | (0·82, 1·41) | 0·6 |
| G2 (N=1349) | 1·07 | (0·91, 1·26) | 0·4 | 1·18 | (0·91, 1·52) | 0·2 |
| G3 (N=1442)) | 1·06 | (0·90, 1·23) | 0·5 | 1·02 | (0·79, 1·31) | 0·9 |
| G4 (N=1453) | 1·00 |  |  |  |  |  |
| **Prospective (N=6057)** |  |  |  |  |  |  |
| G1 (N=1776) | 1·07 | (0·91, 1·27) | 0·5 | 1·28 | (1·04, 1·58) | 0·02 |
| G2 (N=1363) | 1·02 | (0·87, 1·20) | 0·8 | 1·10 | (0·89, 1·36) | 0·4 |
| G3 (N=1456) | 1·09 | (0·93, 1·27) | 0·3 | 1·10 | (0·89, 1·34) | 0·4 |
| G4 (N=1462) | 1·00 |  |  | 1·00 |  |  |
| **Work Activity Only** |  |  |  |  |  |  |
| **Cross-sectional (N=2756)** |  |  |  |  |  |  |
| G1 (N=590) | 0·49 | (0·38, 0·63) | <0·001 | 0·45 | (0·33, 0·63) | <0·001 |
| G2 (N=588) | 0·70 | (0·55, 0·88) | 0·002 | 0·71 | (0·54, 0·94) | 0·02 |
| G3 (N=716) | 0·91 | (0·74, 1·12) | 0·4 | 0·86 | (0·67, 1·09) | 0·2 |
| G4 (N=862) | 1·00 |  |  | 1·00 |  |  |
| **Prospective (N=5020)** |  |  |  |  |  |  |
| G1 (N=1338) | 0·57 | (0·47, 0·69) | <0·001 | 0·59 | (0·47, 0·74) | <0·001 |
| G2 (N=1091) | 0·74 | (0·62, 0·89) | 0·001 | 0·74 | (0·60, 0·91) | 0·01 |
| G3 (N=1271) | 0·94 | (0·80, 1·11) | 0·5 | 0·94 | (0·77, 1·13) | 0·5 |
| G4 (N=1320) | 1·00 |  |  | 1·00 |  |  |

For most of the tests, the overall relationship using groups of approximate quartiles was not different from that as observed in the main analyses (as shown in Table 3 ) using the top and bottom tenth percentiles. There was little or no association between habitual inactivity and cognition. Differences were observed for VST-simple using approximate quartiles, where being physically inactive increased the risk of being in the lowest performance group (OR=1.17 (95%CI 1.01, 1.36) P=0.03)). This was not observed using the more stringent bottom tenth percentile. This is likely due to lack of statistical power. For VST-complex, the OR were attenuated in the supplementary analyses (with the larger groups) and no longer statistically robust. Choosing an appropriate percentile to represent abnormality for cognition can vary across tests. Changing the grouping of the cognitive measures in this relatively healthy cohort of men and women did not change the overall findings of the main analysis using more stringent cut-off.

**Appendix 1**

**Work and Leisure time components of the EPIC Physical activity questions from which total physical activity score derived**

1. We would like to know the type and amount of physical activity involved in your work. Please tick what best corresponds to your present activities from the following four possibilities:

Sedentary occupation - You spend most of your time sitting (such as in an office)

Standing occupation - You spend most of your time standing or walking. However, your work does not require intense physical efforts (e.g. shop assistant, hairdresser, guard, etc.)

Physical work - This involves some physical effort including handling of heavy objects and use of tools (e.g. plumber, cleaner, nurse, sports instructor, electrician, carpenter, etc.)

Heavy manual work - This involves very vigorous physical activity including handling of very heavy objects (e.g. docker, miner, bricklayer, construction worker, etc.)

1. In a typical week during the past 12 months, how many hours did you spend on each of the following activities? (Put ‘0’ if none) Cycling, including cycling to work and during leisure time

In summer _______ hours per week

In winter _______ hours per week

Other physical exercise such as keep fit, aerobics, swimming, jogging

In summer _______ hours per week

In winter _______ hours per week

Hours per day of recreational activity computed from [(mean of summer and winter hours per week cycling) + (mean of summer and winter hours per week other physical exercise)]/7.

**Appendix 2**

The four levels of the index are as follows:

(1) Inactive (a sedentary job and no recreational activity)

(2) Moderately inactive (a sedentary job with, 0·5 h recreational activity per day or standing job with no recreational activity)

(3) Moderately active (sedentary job with 0·5–1 h recreational activity per day, or standing job with, 0·5 h recreational activity per day, or physical job with no recreational activity)

(4) Active (sedentary job with 1h recreational activity per day, or standing job with 1h recreational activity per day, or physical job with at least some recreational activity, or heavy manual job).

**Appendix 3**

**Creating the cognition composite score from the EPIC-Norfolk Cognition Battery (EPIC-COGComp)**

**Method**

A composite score (EPIC-COGComp), in theory should be a stronger measure of overall cognition than any individual item. EPIC-COGComp underlies all the cognitive abilities covered in the EPIC-Norfolk Cognition Battery.

For each of the individual cognition test with a continuous score, three groups were generated as follows:

**Poor Performance**: Participants were classified to this group if they obtaining a score less than a cut-off point corresponding to approximately the 10th percentile of the population distribution. Poor performance was assigned a score of 0.

**High Performance**: Participants were classified to this group if they obtaining a score above a cut-off point corresponding to approximately the 90th percentile of the population distribution. High performance was assigned a score of 2.

**Standard Performance:** All remaining participants not within the bottom or top tenth percentile groups were classified to this group. Standard performance was assigned a score of 1.

For prospective memory, where participants either succeeded or failed the task, this variable was dichotomised. Those failing the task were classified to the poor performance group and assigned a score of 0 and those who were successful were assigned a score of 1.

**The EPIC-COGComp** composite score was calculated as a sum of the score based on the performance group for all eight cognition test outcomes (EPIC-COGComp score range 0-15). The lowest score was 0 (being in the poor performance group for all 8 cognitive test outcomes), although only 4 participants in the cohort had a score of 0. The highest score in the cohort was 14, with no participant attaining the top score of 15 (being in the high performance group for all 8 cognitive test outcomes).

A three–level categorical variable of the EPIC-COGComp score was created from the continuous score using the bottom 10^th^ percentile (obtaining a score of 6 or below), top 10^th^ percentile (obtaining a score of 10 or above) and those with scores between 7-9 in the standard performance group.

**Creating groups for Supplementary analyses**

Four groups (that approximate quartiles of the population distribution in each of the eight cognitive outcome measures) were generated (G1-G4). The highest performance group (G4) was the reference group. The approximate quartile group composite score was as above, the sum of the score based on the performance group for all eight cognition test outcomes (score range 0-22). The lowest score was 0 (being in the lowest approximate quartile for all 8 cognitive test outcomes), with 22 participants in this group. There were 9 participants who were in the top approximate quartile and successful in the prospective memory task obtaining the top score of 22.
